# Interdisciplinary tutorial. Skills lab training in oral health as a strategy for promoting interdisciplinary skills

**DOI:** 10.3205/zma001747

**Published:** 2025-04-15

**Authors:** Nima Gholamzadeh Biji, Marc André Ackermann, Florian Lautenbacher, Susanne Borgmann, Sabine Sennhenn-Kirchner, Iris Demmer

**Affiliations:** 1 University Medical Center Göttingen, Department of Oral and Maxillofacial Surgery, Göttingen, Germany; 2 University Medical Center Göttingen, Study Deanery of the Medical Faculty, STÄPS, Göttingen, Germany

**Keywords:** skills lab, oral pathologies, oral health, interdisciplinary knowledge, peer teaching, intraoral diagnosis

## Abstract

**Objective:**

Trained student tutors instruct medical and dentistry students in the methodology of structured intraoral diagnosis, the identification of common oral pathologies, and the fundamental treatment concepts. The objective of this study is to assess the feasibility and efficacy of a peer teaching-based interdisciplinary tutorial and a training program for tutors designed for this purpose.

**Methods:**

The present study describes the feasibility, evaluation, and implementation of an interdisciplinary tutorial for medical and dental students at the University Medical Center Göttingen. The development of tutorial contents, monitored by experts, and structured training of the tutors formed the foundation for the implementation of the tutorial. The concepts for the tutor training and the tutorial were evaluated by the participants. The efficacy of the tutorial in enhancing the participants’ clinical skills was assessed through analysis of their performance in a mini-clinical evaluation exercise on intraoral diagnosis and a mini-quiz comprising nine questions.

**Results:**

The structured training program enabled tutors to enhance their theoretical and practical abilities, as well as their educational competencies. The peer teaching tutorial, conducted in accordance with the training program, facilitated the advancement of the participating students’ clinical knowledge and practical skills.

**Conclusion:**

The interdisciplinary student tutorial equipped medical and dentistry students with pertinent interdisciplinary competencies drawn from the medical and dental learning objectives catalogues on oral health topics, thereby raising awareness of their future relevance in the context of medical and dental practice. Both the student tutors and the students participating in the tutorial benefited from the educational approaches delineated as part of the study. Other medical skills laboratory teams may use and adapt these approaches.

## Zusammenfassung

**Zielsetzung:**

Geschulte studentische Tutor:innen vermitteln Humanmedizinstudierenden und Zahnmedizinstudierenden die Erhebung eines strukturierten intraoralen Befundes, die Identifizierung häufiger oraler Pathologien und grundlegende Behandlungskonzepte. Ziel dieser Studie ist es, die Umsetzbarkeit und Wirksamkeit eines peer teaching-basierten interdisziplinären Tutoriums und eines dafür erstellten Schulungskonzeptes für Tutor*innen zu evaluieren.

**Methoden:**

In dieser Studie wurde ein interdisziplinäres studentisches Tutorium für Medizinstudierende und Zahnmedizinstudierende der Universitätsmedizin Göttingen pilotiert, evaluiert und implementiert. Die durch Expert*innen supervidierte Erarbeitung der Tutoriumsinhalte und eine strukturierte Schulung der Tutor:innen bildeten die Grundlage für die Umsetzung des Tutoriums. Die Konzepte für die Tutor:innenschulung und für das Tutorium wurden von den Teilnehmenden evaluiert. Der Lernerfolg des Tutoriums auf klinische Kompetenzen der Teilnehmenden wurde anhand der Ergebnisse einer Mini-Clinical Evaluation Exercise zur intraoralen Befunderhebung und eines Mini-Quiz mit neun Fragen erfasst.

**Ergebnisse:**

In einer strukturierten Schulung konnten Tutor:innen ihr Wissen, praktische Fertigkeiten und Lehrkompetenzen verbessern. Das auf dieser Grundlage durchgeführte peer teaching-Tutorium verbesserte klinisches Wissen und praktische Fertigkeiten der teilnehmenden Studierenden.

**Schlussfolgerung:**

Das interdisziplinäre studentische Tutorium vermittelte Studierenden der Humanmedizin und der Zahnmedizin relevante fächerübergreifende Kompetenzen der medizinischen und zahnmedizinischen Lernzielkataloge zu Themen oraler Gesundheit und sensibilisierte für deren Relevanz in der zukünftigen ärztlichen und zahnärztlichen Tätigkeit. Sowohl die studentischen Tutor:innen als auch die am Tutorium teilnehmenden Studierenden profitierten von den im Rahmen der Studie etablierten Lehrkonzepten und andere medizinische Skills-Lab-Teams können diese nutzen und anpassen.

## 1. Introduction

During their daily practice, physicians and dentists are frequently confronted with the task of recording intraoral diagnosis and categorizing them in the context of a systemic disease or a specific complaint. It is therefore essential that they possess both a structured procedure and a comprehensive understanding of relevant oral pathologies in order to collect and interpret intraoral diagnosis in an accurate and systematic manner [1], [2].

The National Competence-Based Catalogue of Learning Objectives in Medicine 2015 (NKLM) outlines the fundamental clinical practical competencies and explicit learning objectives related to oral health for the clinical study section. The emphasis is on acquiring knowledge of the structure of oropharyngeal structures and their clinical examination [https://nklm.de/menu], [http://www.nklz.de/kataloge/nklz/lernziel/uebersicht]. In addition to the development of specific dental skills, clinical dental training incorporates an examination of general medical factors and the influence of dental pathologies on general health [http://www.nklz.de/kataloge/nklz/lernziel/uebersicht]. In accordance with §3 of the recently introduced dental licensing regulations, there is a clear emphasis on fostering interdisciplinary thinking among prospective dentists, particularly in the future [https://www.gesetze-im-internet.de/zappro/__3.html].

The necessity for interdisciplinary training concepts for medical and dentistry students in Germany has already been demonstrated [3]. Prior studies have indicated the beneficial impact of interdisciplinary knowledge transfer [4], [5], [6]. Peer teaching, also referred to as peer-assisted learning, has been demonstrated to be as effective as medically instructed training [7], [8]. The congruence between the social and cognitive abilities of the student tutors and those of the participating students is a significant factor in determining the effectiveness of the tutoring process [9], [10]. To date, there is no existing project in the academic literature that provides a peer teaching-based tutorial for students enrolled in both degree programs, which addresses the topic of promoting oral health and identifying oral pathologies. Furthermore, there has been no research on structured training of medical and dental students as part of interdisciplinary tutorials [3].

The Dentistry degree program at the University Medical Center Göttingen (UMG) places an emphasis on intraoral diagnosis from the second clinical semester onwards. This forms the foundation for the subsequent clinical teaching and is also the basis for student treatment of patients during the course of study [http://www.nklz.de/kataloge/nklz/lernziel/uebersicht], [https://www.gesetze-im-internet.de/zappro/__3.html]. The dental aspects of intraoral diagnosis are taught in lectures and compulsory seminars during the fourth clinical semester of medical education at the UMG and are examined as part of a module examination. There is no routine practical application of intraoral diagnosis. Inadequate intraoral diagnosis in clinical training and patient care can impair targeted prevention and differential diagnosis [11], [12].

Against this background, an extracurricular interdisciplinary tutorial on oral pathologies was initiated in collaboration with the medical skills lab STÄPS (Studentisches Trainingszentrum ärztlicher Praxis und Simulation [https://www.umg.eu/studium-lehre/staeps/]) and the dental medicine skills lab SINUZ (Studentisches Innovations- und Trainingszentrum Zahnmedizin [https://sinuz-goettingen.de/]) targeting complementing the curricular and dental medicine training at the UMG [13].

The tutorial is designed to equip medical and dentistry students with an enhanced understanding of oral health, an awareness of oral pathologies and proficiency in intraoral diagnosis through a comprehensive training approach.

The aim of this project is to describe the development, implementation, and evaluation of an interdisciplinary tutorial on oral health. Additionally, a training program for tutors was established, implemented, and evaluated. This training is considered to equip tutors with the requisite knowledge and skills to effectively impart oral health information and to record intraoral diagnosis.

## 2. Methods

### Study design

The peer teaching-based teaching project presented here was designed as a monocentric, uncontrolled, prospective study for medical and dentistry students at the UMG. The project is based on the concept of the skills lab, in which structured, supervised learning takes place through the use of student tutors and the implementation of appropriate didactic approaches [8], [14].

The study involved three distinct groups of individuals: the study participants (hereafter referred to as “participants”), the student employees of the two skills labs (referred to as “tutors”), and the supervisors. All interested student tutors from both skills labs were eligible to participate in the tutor training program. The project was monitored by a team of qualified professionals, including dentists (authors SSK, head of SINUZ, NGB, MA, FL), a specialist in general practice (author ID, head of STÄPS), and a pedagogue (author SB, STÄPS employee).

From the winter semester of 2019/20 until the initiation of the study, interested student tutors of STÄPS and SINUZ from the UMG collaborated with the supervisors to develop the content of the interdisciplinary tutorial, with a particular focus on oral health and intraoral diseases. The topics were identified and prioritized for integration into the tutorial in a two-hour face-to-face meeting of the authors, utilizing the nominal group technique [15]. To guarantee the quality of the practical units and structured feedback, it was necessary to provide two-to-one supervision of the participants by the tutors.

The tutorial was designed for medical and dentistry students who were engaged in clinical study phases. Participation was open to all students, with no restrictions. The study was conducted as part of the tutorial during the winter semester of 2022/2023 and the summer semester of 2023. The tutorial was advertised from the summer semester of 2022 during the lecture period via oral communication, Instagram presences of the skills labs and semester-internal messenger groups. One week prior to the beginning of the tutorial, the participants were informed about the study and the voluntary participation in the anonymized data collection and evaluation of the project. The initial pilot of the tutorial was conducted on 15 June 2022.

### Tutor training

As part of the tutorial concept, a training course for tutors was developed to facilitate the acquisition of interdisciplinary knowledge and skills for the effective teaching of the tutorial. Students of medicine and dentistry were recruited as tutors. The creation of a standardized knowledge base for the tutorial’s content should also facilitate the provision of competent support by students of other subject areas.

**Figure 1:**
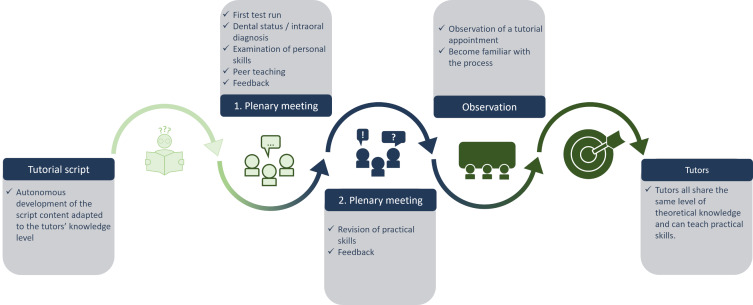
Procedure for the tutor training

A tutorial script was established by one of the supervisors (author NGB, see figure 1 (Fig. 1)). It contains information on the premises and the course of the tutorial, lists of materials, learning objectives and knowledge inputs on three topic blocks of the tutorial as well as didactic tips for the tutors.

In the initial one-hour session, open questions were elucidated and selected topics were discussed in plenary. After dental supervision, two tutors took each other's intraoral diagnosis and evaluated their practical skills. At the conclusion of the first session, the dental supervisors presented a comprehensive schematic representation of the intraoral diagnosis.

The second tutor training session, which occurred one week later, comprised a one-hour review of the previously discussed practical procedures for intraoral diagnosis. This was done to assess the extent to which the tutors had retained the knowledge gained in the first session. Additionally, the tutors received feedback from one of the supervisors after the second session. This was done to evaluate the tutors’ learning success and implement any final improvements.

The final stage of the training program comprised a work shadowing session at a tutorial. This enabled the tutors to become acquainted with the tutoring process and to reinforce their theoretical understanding of the content and practical skills.

### Procedure of the tutorial and interventions

The tutorial, entitled “Oral Pathologies”, was conducted during the winter semester of 2022/23 and the summer semester of 2023. The number of participants ranged from six to ten students.

**Figure 2:**
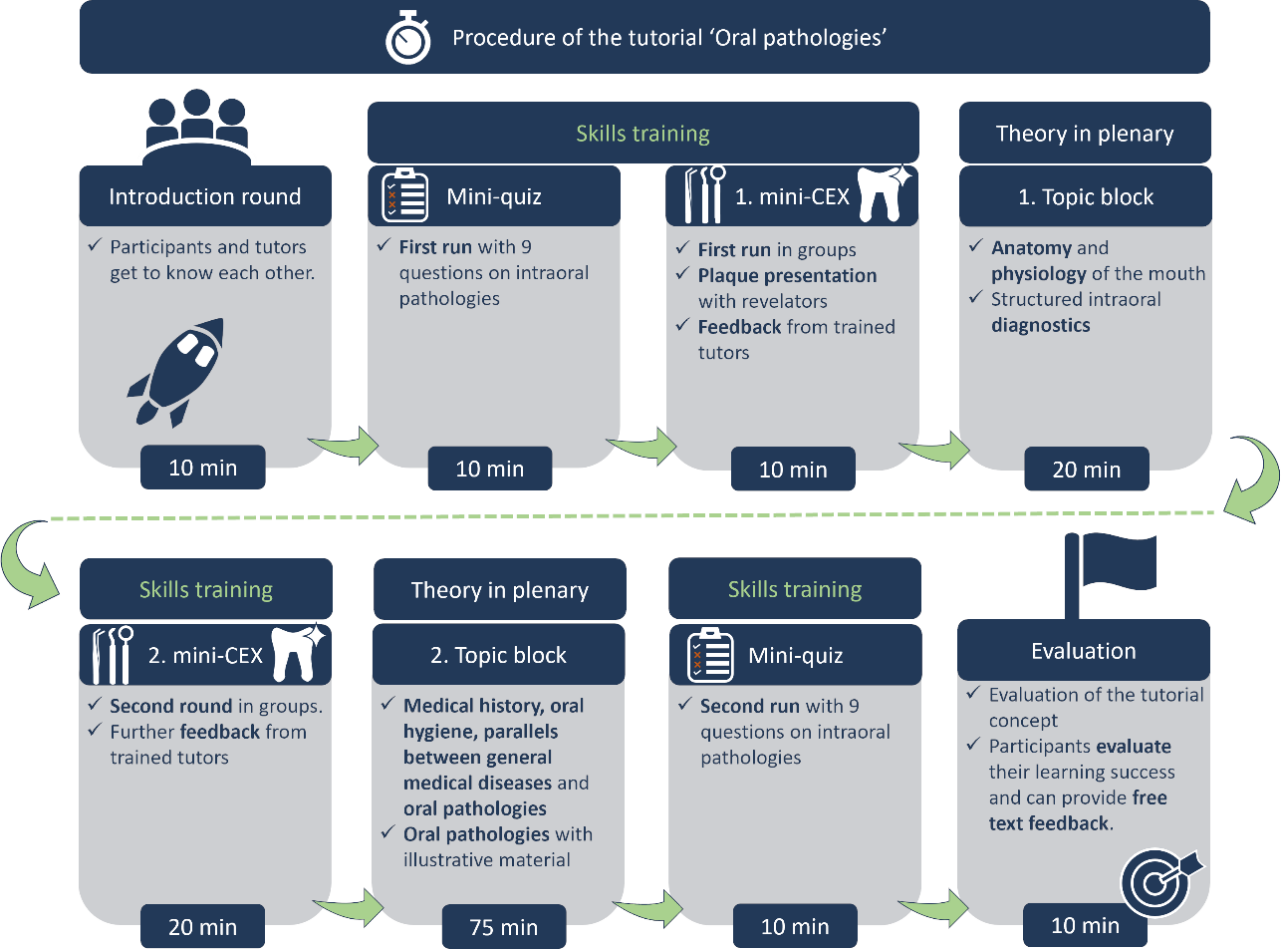
Procedure of the tutorial “Oral Pathologies”

Following a brief introduction of the participants and tutors, the tutorial proceeded with a mini-quiz (see figure 2 (Fig. 2)). The assessment comprised nine multiple-choice questions on the principal topics of the tutorial, with one point awarded for each correct answer. The responses to each question were documented and subjected to analysis using the web-based evaluation software evasys [https://evasys.de/en/].

Subsequently, the participants were tasked with conducting an intraoral examination without prior instruction. One tutor was assigned to supervise two participants at a time, with each participant taking turns to perform a finding on the other.

In order to establish an objective measure of learning success, both the tutors undergoing training and the participants were required to complete a mini-Clinical Evaluation Exercise (mini-CEX) prior to and following the training or tutorial [16]. The objective was to evaluate each other's intraoral diagnosis in pairs. The examination was conducted in the dental treatment unit of the student skills laboratory SINUZ with the use of an oral spatula and mouth mirror. Based on eleven criteria with a three-level rating scale, oral feedback was provided to the tutors by the team dentists (NGB, MA, FL) and to the participants by the tutors trained during the course. The evaluations were saved and analyzed using the web-based evaluation software evasys [https://evasys.de/en/].

Following the initial mini-CEX, the participants in the tutorial were subjected to their inaugural intervention, which comprised a discussion of the anatomical structures of the oropharyngeal system and dental diagnosis. These were developed collectively and interactively with the objective of standardizing the theoretical principles of intraoral diagnosis. The interventions were concluded with an exemplary and comprehensive demonstration of the intraoral diagnosis discussed by the tutors. The participants were then permitted to undertake another intraoral assessment independently, with the opportunity to receive feedback from a tutor.

The second intervention for the participants was conducted following the second mini-CEX and comprised instructions on the maintenance of appropriate oral hygiene. The objective was to instruct the participants on the correct presentation and utilization of pertinent oral hygiene products. To emphasize the significance of adequate oral hygiene for all medical specialties, the parallels between pathological alterations in the oral cavity and general diseases were then elucidated. In addition, the participants had the opportunity to learn about the most important benign and malignant oral mucosal changes and tested their knowledge by repeating the mini-quiz.

### Outcome parameters

The training provided to tutors was evaluated to ascertain the extent to which it had enabled them to develop their knowledge and skills. In addition, the effectiveness of the training concept was assessed. Regarding the tutoring program itself, the learning growth of the participants was evaluated, as was the quality of the tutoring program.

### Evaluation 

**Figure 3:**
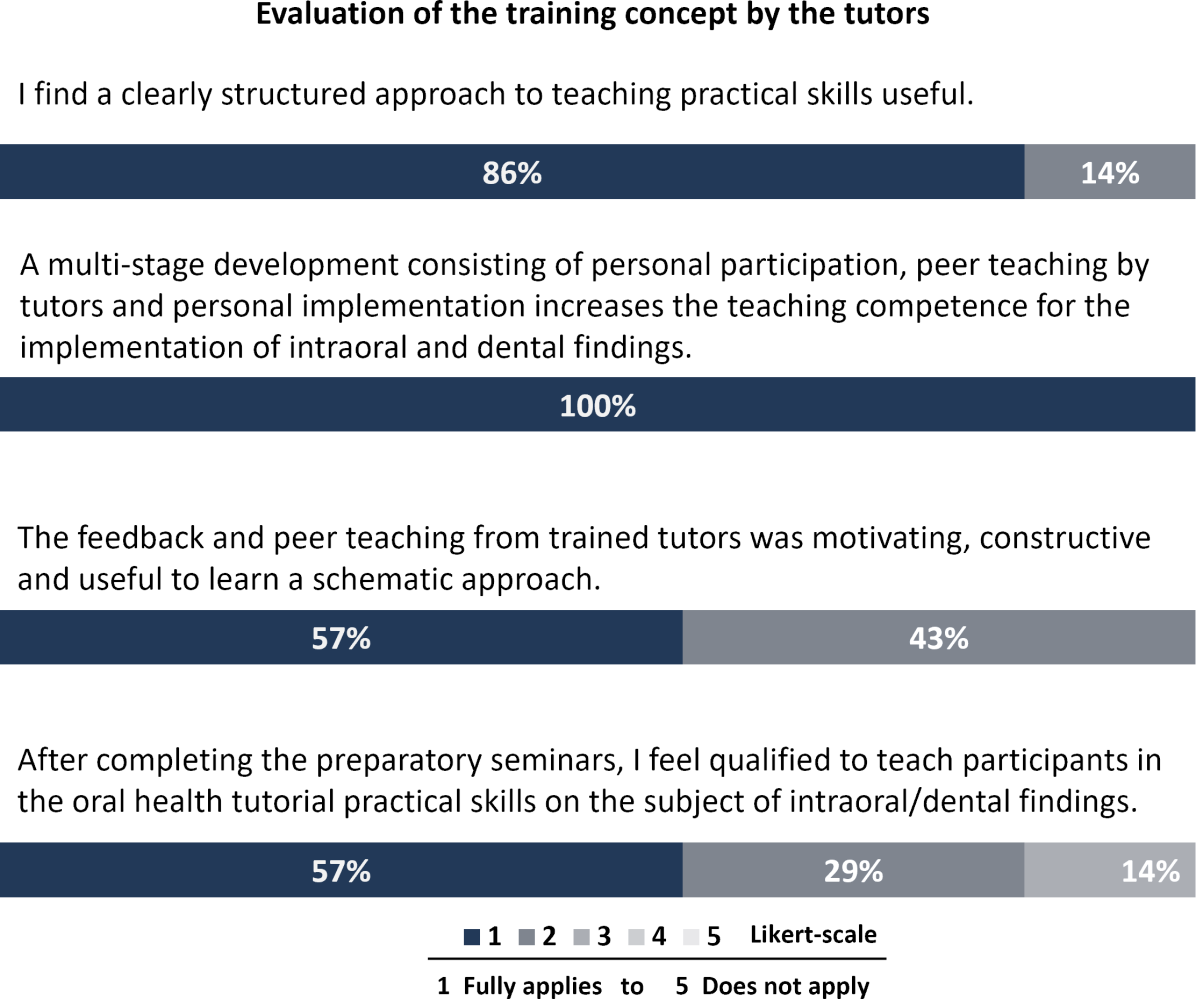
Evaluation of the training concept by the tutors

To evaluate the efficacy of the tutor training course, the tutors were asked to assess themselves after the course in terms of the practical skills they had acquired for the role of tutor. They were also invited to evaluate the training concept in terms of the following two areas: didactics (comprising three items) and a further overarching self-assessment of learning growth (comprising one item, each on a five-point Likert scale) (see figure 3 (Fig. 3)).

**Figure 4:**
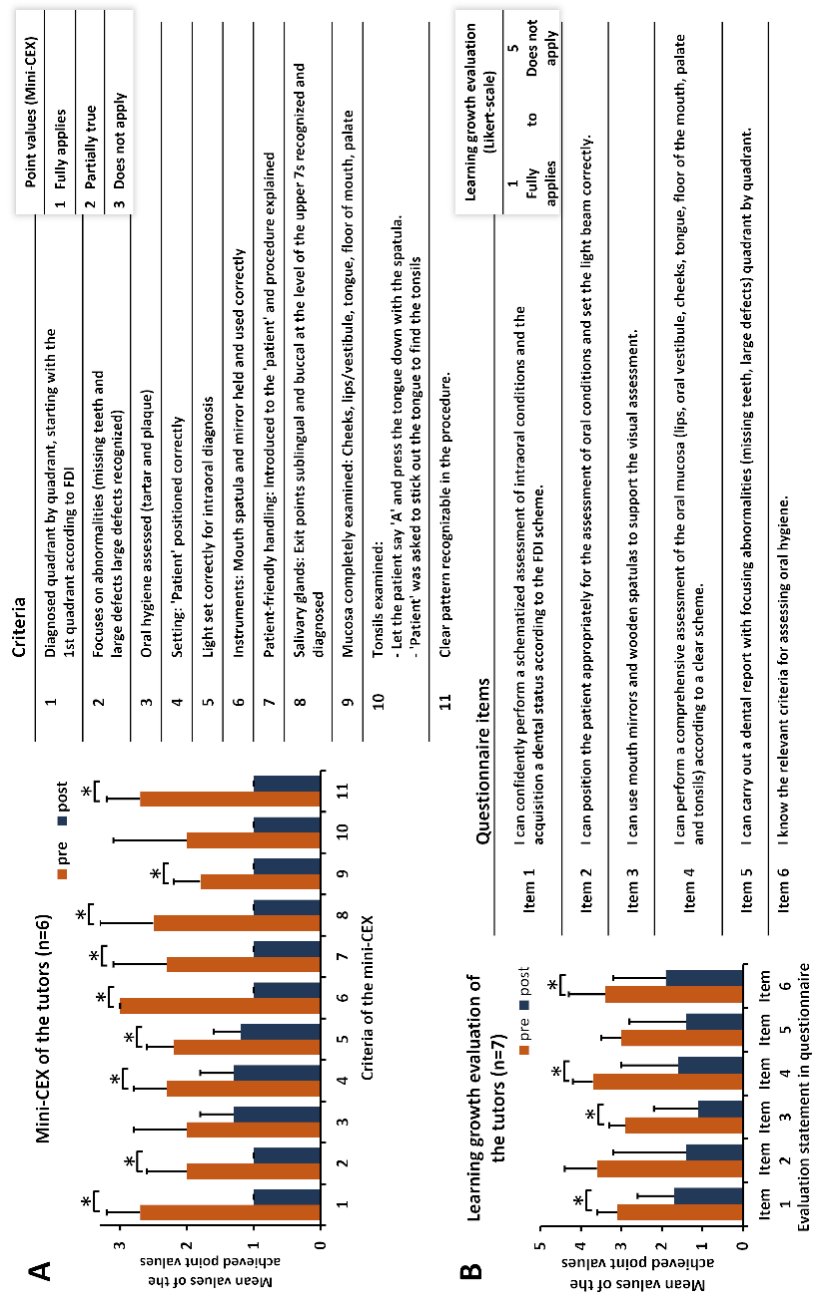
Results of the mini-CEX and learning growth evaluation of the tutors

The tutorial was evaluated by participants via a written pre- and post-self-assessment of their learning growth for five learning objectives, utilizing a five-point Likert scale. The quality of the tutor training was assessed by the tutors regarding the framework conditions, the didactic concept, the learning atmosphere, and knowledge transfer, using a five-point Likert scale (see figure 4 (Fig. 4)).

### Data collection/data protection

The results of the mini-CEX and mini-quiz were stored and subsequently subjected to analysis in an anonymized form.

In accordance with Article 13 of the GDPR [https://gdpr-info.eu/art-13-gdpr/], the tutors and participants were duly informed and consented to the collection of data for the mini-CEX and mini-quiz, respectively. The data was collected electronically using evasys 9.1 [https://evasys.de/en/] for the mini-CEX and via “ILIAS open-source e-Learning e.V. ” [https://www.ilias.de/en/] for the mini-quiz.

The participants were granted access to the mini-quiz via their individual Stud.IP profiles and provided with a link to ILIAS. In the instance of personal data collection (mini-quiz), anonymization was conducted prior to the commencement of data processing [17].

### Statistics

A pre-post comparison was conducted to determine whether the data exhibited a significant improvement, employing the Mann-Whitney U-test for this purpose [18], [19]. All data were analyzed using the statistical software package SPSS (version 29.0) and p-values less than 0.05 were statistically significant. The results that reached this level of statistical significance were labelled with an asterisk in the figures.

### Ethics vote

The ethics committee of Georg-August University has granted approval for the project (reference number: 15/12/22).

## 3. Results

### Evaluation by the tutors

All tutors (n=7) participated in the evaluation of the training concept for tutors. The tutors demonstrated near-unanimous agreement that a structured approach and multi-stage development (participation, peer teaching, and independent implementation) can facilitate an increase in teaching competence. The peer teaching was predominantly (57%) perceived as motivating, constructive and helpful by the tutors. Following the training, 86% of tutors felt fully or mostly competent to teach practical skills to students on oral health and intraoral assessment (see figure 3 (Fig. 3)).

### Learning growth of the tutors

Figure 4 (Fig. 4) presents a summary of the tutors’ learning growth. In the objective study of learning growth using mini-CEX (n=6), the pre-post comparison demonstrated a significant learning growth in nine out of 11 criteria between the initial and subsequent performances of the intraoral diagnosis (see figure 4 A (Fig. 4)). The learning growth in the criteria “Assessment of oral hygiene” and “Complete diagnosis of the mucosa” did not demonstrate any notable improvements.

The self-assessed learning growth of the tutors (n=7) was reflected in the pre-post comparison, with a significant improvement observed in four out of six competence topics. However, no significant learning growths were noted for the topics “advantageous patient positioning with light cone setting” and “oral diagnosis with a focus on abnormalities” (see figure 4 B (Fig. 4)).

### Evaluation by participants

**Figure 5:**
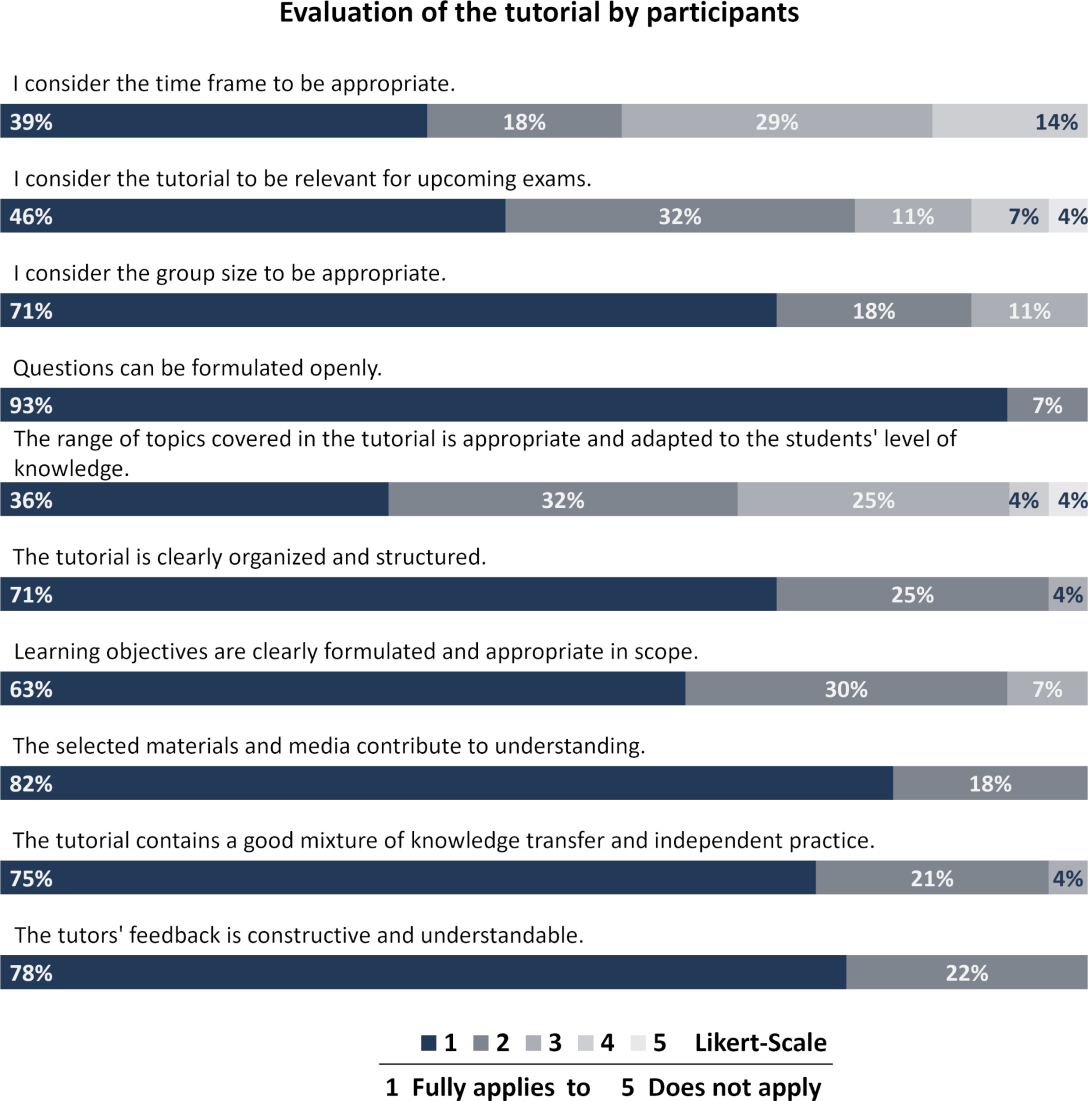
Evaluation of the tutorial by the participants

The tutorial was evaluated by the participants (n=28) in 10 aspects (5 framework conditions, 5 content aspects) using a five-point Likert-scale (see figure 5 (Fig. 5)). In terms of the framework conditions, the participants expressed high levels of agreement with several aspects, including the appropriate group size (71% f.a. (full agreement)), the mixture of knowledge transfer and independent practice (75% f.a.), the clear structure of the tutorial (71% f.a.) and the opportunity to formulate open questions (93% f.a.). A mere 39% of respondents rated the time frame as completely sufficient.

In terms of content, participants responded positively to the constructive and understandable feedback provided by tutors (78% f.a.), the clearly formulated and appropriate learning objectives (63% f.a.) and the effectiveness of the selected materials in facilitating understanding (82% f.a.). However, the relevance of the tutorial to examinations (46% f.a.) and its adaptation to students’ varying levels of knowledge (36% f.a.) were reflected less positively in the evaluation.

### Learning growth of the participants

**Figure 6:**
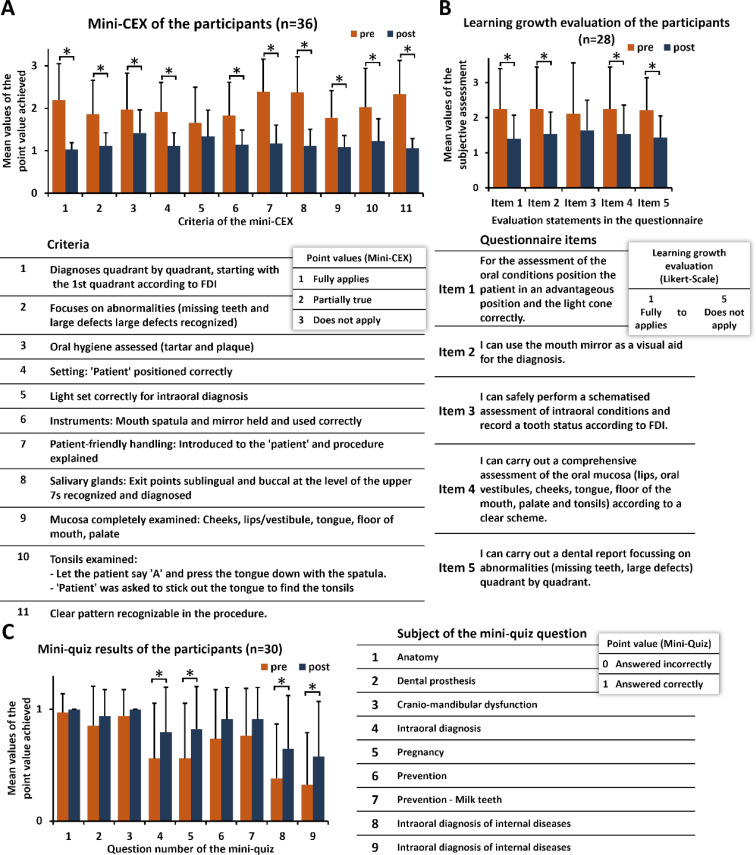
Results of the mini-CEX and learning growth evaluation of the participants

A total of 36 participants completed the mutual assessment in a mini-CEX under the observation of the tutors. All participants were assessed in both rounds. The intraoral inspection, conducted under the supervision of the tutors, considered and evaluated eleven criteria (see figure 6 A (Fig. 6)). The intraoral assessment demonstrated improvement for all participants between the first and second runs. Significant changes were observed for ten of the eleven criteria between the first and second performance of the intraoral diagnosis, apart from “correct light setting for oral diagnosis”, which did not reach statistical significance.

The learning growth evaluation of the participants was conducted using six defined items, which demonstrated a notable enhancement in the self-assessed practical skills of the participants (n=28) across four out of five aspects (see figure 6 B (Fig. 6)). In the self-assessment of intraoral conditions and dental status according to the Fédération Dentaire Internationale (FDI), the participants indicated that they did not perceive an increase in confidence compared to their pre-tutorial level.

A mini-quiz conducted before and after the tutorial (n=30) demonstrated that the proportion of correct answers to all nine topics was higher after the tutorial (see figure 6 C (Fig. 6)). However, the increase in correct answers was only statistically significant for four of the nine questions, namely those pertaining to intraoral diagnosis, pregnancy and two instances of intraoral diagnosis of internal diseases.

## 4. Discussion

This study presents the piloting of an interdisciplinary tutorial that enables medical and dentistry students to exchange relevant knowledge on oral pathologies and to implement this knowledge in the form of a targeted intraoral diagnosis. The objective of the study was to demonstrate the appropriate feasibility of the student tutorial and the positive effect for participating students from both disciplines. Furthermore, a training concept was established through which tutors from both disciplines can acquire equivalent theoretical knowledge and practical skills. This concept is intended to enable tutors to teach theoretical and practical principles. Additionally, a script that can be passed on to subsequent generations of tutors is intended to ensure the sustainable quality of subsequent tutorials.

The evaluation data collected allows us to conclude that the implementation of the tutorial was a success. The participants expressed high levels of satisfaction with the tutorial’s concept and structure. The tutorial, which was designed for medical and dentistry students, addressed the topic of oral health and pathologies. It was based on peer teaching and proved effective in consolidating relevant knowledge. This was achieved through repeated test simulations, which were followed by feedback in the form of the mini-CEX and the mini-quiz [20]. The established training procedure allows for the introduction of tutors from outside the subject area to specific theoretical and practical content, thus ensuring their ability to lead and supervise the tutorial in a safe manner. However, the initial organization of the preparation of all tutors and the creation of various assessment criteria and preparation of practical exercises for the skills training represents a significant undertaking. To implement such a project, it is essential that preparatory work is carried out by dentists at the outset. Once the initial cohort of tutors has undergone training and all the requisite criteria for skills training, including digital evaluation forms, have been established, the effort required to continue the tutorial will have been reduced to a level commensurate with that of peer teaching-based projects.

The tutors’ evaluation data on their training indicates that structured preparation for teaching practical skills based on standardized criteria is perceived as useful and helpful. In particular, the multi-stage structure of the concept, in conjunction with the provision of constructive feedback from previously trained tutors on two occasions, was rated as an effective preparation for the practical elements of the tutorial. Following the completion of the training, most tutors felt confident in their ability to teach both theoretical and practical skills on the topic of intraoral and dental diagnosis for the tutorials. The impact of this training concept was reflected in the positive evaluations provided by the participants.

Furthermore, the results of the tutors’ mini-CEX in the pre-post comparison of their practical performance in intraoral diagnosis demonstrate a notable enhancement in the tutors’ proficiency across all sub-areas of the assessment criteria. This demonstrates that the structured training program has a beneficial impact on the tutors’ acquisition of practical competencies. A fundamental requirement for the success of an interdisciplinary tutorial involving tutors from diverse subject areas and with varying levels of prior knowledge is to establish an approximately equivalent foundation of knowledge and skills for all tutors. As a valuable supplement to the mini-CEX results, the tutors’ self-assessed improvements in the assessment criteria following their participation in the training courses also provide a suitable addition to the evaluation process [21]. The results consistently demonstrate that the implementation of tutor training has a positive impact on the ability to clearly structure an intraoral finding and to apply the associated practical skills.

In view of the restricted resources available, the study was obliged to be conducted in a non-controlled manner. The focus of this work was the development and establishment of an interdisciplinary tutorial as preliminary work for a possible controlled study later. In addition to the interventions, which included imparting knowledge and practicing intraoral diagnosis, the mini-quiz and the mini-CEX as pre-post-tests also had positive effects on the participants’ and tutors’ learning growths in terms of test-based simulation [22]. With regard to peer teaching-based feedback, the accuracy of peer assessment and feedback should be critically evaluated and given special attention in tutor training [23].

One challenge for the interdisciplinary team of tutors was to establish a uniform level of knowledge across the entire team, which comprised individuals with varying degrees of prior expertise. It was necessary to engage the services of licensed dentists as supervisors for the inaugural tutor training course.

In conclusion, the training concept developed for tutors can be assessed as an appropriate approach for introducing untrained student tutors to the theoretical knowledge and practical skills of an interdisciplinary tutorial.

One objective of the tutorial was to convey as much relevant content as possible on the topic of oral pathologies in one tutorial session. However, during the implementation, it became evident that some participants would have benefited from a more condensed tutorial, enabling them to integrate this extracurricular teaching format into their timetable more effectively. Consequently, the scope of topics was subsequently revised following the study.

In addition to the favorable feedback from participants on this interdisciplinary tutorial, which was collected as free text as part of the learning growth evaluation, the data gathered by the mini-CEX about intraoral diagnosis also corroborates the positive impact of the peer teaching and feedback-based skills training. Most participants demonstrated notable advancement across most diagnostic criteria, attributable to the integration of theoretical knowledge transfer, practical instruction, and oral feedback. The participants indicated that their practical abilities regarding intraoral diagnosis had been enhanced because of their participation in this tutorial. The results of the mini-quiz demonstrated notable advancements in knowledge levels for certain questions following the tutorial. The absence of substantial progress across all subject areas can be attributed to the heterogeneous levels of knowledge among the participants. Consequently, individual learning achievements were observed for specific subject areas.

Overall, the exemplary data collection in the form of mini-CEX and mini-quizzes illustrates the significant benefits of an interdisciplinary tutorial on oral health and oral pathologies.

## 5. Conclusions

An interdisciplinary tutorial, designed and conducted by students, represents an innovative approach to address relevant interdisciplinary knowledge and practical skills for medical and dentistry students. Furthermore, it serves to sensitize these students to the relevance of such knowledge and skills in everyday dental and medical practice. The results of this project demonstrate a beneficial impact of interdisciplinary knowledge transfer on the enhancement of theoretical and practical competencies among participating students and student tutors. To ensure the sustainability of these outcomes, it is essential to provide ongoing supervision by medical and dental professionals and to conduct regular reviews of the tutorial content in alignment with the latest evidence-based findings and recommendations.

## Notes

### Shared authorship

Nima Gholamzadeh Biji and Marc André Ackermann share the first authorship.

### Authors’ ORCIDs

- Nima Gholamzadeh Biji: [0009-0001-7205-5129]
- Marc André Ackermann: [0009-0005-8550-9352]
- Susanne Borgmann: [0000-0003-1570-6068]
- Iris Demmer: [0000-0001-9652-9803]

## Competing interests

The authors declare that they have no competing interests.
